# On the Antidotal Treatment of the Epidemic Cholera; with a Sketch of the Physiology of This Disease, as Deduced from That of Intermittent Fever

**Published:** 1837-01

**Authors:** 


### Art. XII.

1. *On the Antidotal Treatment of the Epidemic Cholera; with a Sketch of the Physiology of this Disease, as deduced from that of Intermittent Fever.* By John Parkin, Member of the Royal College of Surgeons, &c.—*London,* 1836. 8vo. pp. 112.

2. *On the Efficacy of Carbonic Acid Gas in the Diseases of Tropical Climates; with Directions for the Treatment of the Acute and Chronic Stages of Dysentery.* By John Parkin, &c.—*London,* 1836. 8vo. pp. 64.

On several occasions, since the commencement of our labours in this Journal, we have condemned in pretty strong terms the imprudence of young practitioners, of more zeal than discretion, becoming authors before they are masters of any information which it is of importance for the profession to be acquainted with, or even before they have sufficiently disciplined their minds to comprehend the principles on which the sciences of induction and observation are founded. It is with sincere regret that we are compelled to apply the same strictures to the author of the two works before us, because we see in them indications of much industry, zeal, and philanthropy, which promise well for his future success as a practitioner, and perhaps as a writer. A sense of public duty obliges us to state that we hardly recollect to have seen compressed within the same extent of space so much unfounded hypothesis, gratuitous assumption, and illogical inference, redeemed by so little matter of fact of any value.

In reading the exposition of Mr. Parkin's theory of the development of cholera, we are ever and anon tempted to think that we are perusing some of the speculations of our bookish predecessors before the Baconian philosophy had put a check to the fantasies of learned dreamers, or the discoveries of modern physiology had given some form and consistency to the observations and enquiries of medical men.

The accommodating nature of Mr. Parkin's morbific poison (malaria,) when once introduced into the system, and the no less ready ministration of the fluid (the blood) which transports it so carefully from one place to another at the precise time required by the theory, as well as its capacity to produce all the requisite phenomena, must strike every one, no less that the simplicity of the author, who never for a moment seems to be aware of the necessity of adducing some *proofs* of the existence of his poison within the body, and of its peregrinations and *modus operandi* while there.

The following passages which we extract from the section entitled "Physiology," (qy. Pathology,) will sufficiently indicate the æra of the school to which the author belongs. We ought to observe that some of the passages we are going to quote refer to intermittent fever and not cholera; but, as both are regarded as flowing from the same "almost universal cause" of disease, *malaria*, and as the phenomena in ague are stated to be "similar in every respect to those of cholera asphyxia," the theory is equally applicable to both diseases.

"Taking it for granted, then, that the atmosphere is the vehicle for the introduction of the poison into the system, we may also infer that it first enters the lungs, and then passes on through the left side of the heart into the divisions and subdivisions of the larger arteries, until ultimately it reaches their extreme termination—the capillary vessels. But, although it enters the arterial portion of the circulating system, we cannot suppose that it remains long or to any extent in this situation." . . . . "We may therefore conclude, that the accumulation of the poison, after its first introduction into the human frame, and before it produces any specific effect, does not take place in the arterial system. But if not in the arterial branches of the circulating system, where then does the poison accumulate? As there are but three divisions in this circle, if the poison passes into the circulating mass, and if it does not remain in the arterial, we cannot err much in inferring that the accumulation takes place either in the venous or the pulmonic system." . . . . "If, then, we suppose that the poison has been propelled into the extreme divisions or subdivisions of the veins, it is allowable to conclude that, from the operation of physical causes, it will be retained in the venous system for a longer period than in the former instance. Having, however, accumulated to a certain extent, and a given interval having elapsed, it may then pass on with the current of blood into the larger branches, until, ultimately, it reaches the two veins which pour their contents into the right side of the heart. From this situation it may be carried forward by the impetus of the sanguineous current to the lungs; become arrested in the minute capillaries which traverse these organs; escape with the expired air, or enter the pulmonary veins." . . . . "Allowing, for the sake of argument, that the poison passes from the arterial into the venous system when the fever ceases, we will now endeavour to ascertain where this substance is situated when the accession commences. Finding that a certain period elapses, after the cessation of the fever, before any morbid phenomena are again produced, it is reasonable to conclude that, during this interval, the extraneous matter has travelled, with the current of venous blood, from the extremities to the centre." . . . . "If, therefore, we conceive that the malarious agent, having entered the venous system, has been attracted by the operation of laws which seem to regulate the course of extraneous and other matters, from the extremities to the centre, we must also infer that to its detention in this situation, for a certain period, all the morbid phenomena then witnessed are to be ascribed." (P. 5—9.)

The following conclusions terminate the section.

"1. That the epidemic cholera is produced by the introduction of a poison into the system. 2. That the poison in question existed previously in the atmosphere, and entered the lungs with the air inspired. 3. That the accumulation of the poison takes place in the venous system. 4. That in the stage of collapse, the poison is situated in the capillaries of the lungs; and, in the consecutive fever, in those of the skin, as well as other portions of the extreme terminations of the arterial system." (P. 48.)

Without attempting to confute these hypothetical notions, we will merely remark that the most authentic accounts of the distribution of the blood in the collapsed stage of cholera, (see our review of Dr. Mackintosh's work in the present Number,) are at direct variance with them on the matter of fact.

The following extracts will show that Mr. Parkin's principles of cure are as well grounded and equally philosophical as his theory:—

"Having formed a particular theory respecting the epidemic cholera, based on the chemical researches of Dr. John Davy, who ascertained that the expired air of a patient labouring under this disease was deprived of its due portion of carbonic acid, I was induced to administer the different forms of carbon, for the purpose of confirming or refuting the truth of my doctrine. Although now bound to believe that the theory then formed was, in many respects, an incorrect one, it led at least to a not unimportant conclusion—the conviction on my mind that both carbon and carbonic acid remedied the effects witnessed in the epidemic cholera, at the same time that they removed, by their specific action, the cause also." . . . . "A remedy which produces such opposite and different effects, and which has no sensible or direct action in the economy, can only act in one way—that is, by removing the cause of these various phenomena; and as that cause has been proved to be the presence of a poison in the system, the remedy which removes it must neutralize the poison, and must be consequently an antidote in the disease." . . . . "One thing at least is certain, that many poisonous substances, more particularly septic ones, have their injurious properties destroyed or rendered inert when combined either with carbon or carbonic acid. Knowing, therefore, the properties of the various forms of carbon, and observing that, when taken by individuals during an attack of cholera, they instantly arrest the course of the disease, we can only conclude that these agents, when thus given, combine with and neutralize the poisonous matter productive of the effects witnessed in the epidemic cholera." (P. 52—59.)

But, after all, the important question is, not whether Mr. Parkin's doctrines are rational or absurd, but whether he has adduced any evidence to show that his particular mode of practice has cured cholera in times past, or offers a probability of curing it in time to come? And we leave the decision of this question to the reader's judgment, after he has considered the means hitherto employed by Mr. Parkin to overcome this giant malady, and has read the slender catalogue of little facts on which his momentous inferences repose. The essential part of his practice he states to consist in giving the common saline draught (made with half a drachm of carbonate of soda or potass, and a scruple of citric acid,) every hour or every half hour. He says that at the first outset of the attack, "the first dose has always given immediate relief, and the third, at most, removed every symptom," (p. 67;) that, "in the preliminary diarrhœa three or four doses is, in general, sufficient to arrest it," (p. 68;) that, "in the second stage, characterized by rice-water evacuations, the remedy must be given every half hour until the vomiting and purging are entirely arrested," (p. 71;) that, "in the commencement of the stage of collapse, the draught must be given every quarter of an hour, until three or four doses have been taken; then every hour while the purging continues, and every two hours when this has been entirely suspended," (p. 72.) In the two last-mentioned stages, in the more untractable cases, he recommends, in addition to the saline draughts, "pure carbon (charcoal,) either by the mouth or injection," "the inspiration of the gas into the lungs," and even "some stimulant;" the best being "the carbonate of ammonia, either alone or with the acid in effervescence," (p. 71-72.)

These statements imply, either directly or by inference, that Mr. Parkin has cured, and can cure, the Asiatic cholera: and this almost to a certainty by the means indicated. To those who have witnessed any cases of this disease, when epidemic in this or other countries, or who have merely read of the melancholy and hopeless powerlessness of almost all the methods of treatment employed, must assuredly be not a little surprised at Mr. Parkin's confidence and alleged success. Let us see what is the evidence adduced in support of his statements. This is given in the Appendix, and consists partly of cases detailed by Mr. Parkin and others, and partly of fragments of reports from others. Eleven cases are given in all; eight observed by the author; two reported in the Lancet, in 1832, by Mr. Radcliffe, and one treated by Dr. Parkin, of Woolwich, (eight in England, three in Spain.) The majority of these cases were evidently cases of simple diarrhœa, a few seem to have been cases of slight cholera, a few were anomalous, and one of these evidently a case of *fright*. All these cases but one were cured by the saline draughts, some almost instantaneously, all very speedily. The excepted case, that of Dr. Parkin, is represented as "cholera in its severest form," "although no vomiting or purging was observed previous to the attack;" and this was cured instantaneously by a single draught of brisk bottled porter!

The second class of documents consists of short fragments of letters and reports from three or four Spanish physicians, who, during the prevalence of the epidemic cholera in Spain, administered the saline draughts, by the recommendation of Mr. Parkin. It is to be regretted that no statistical details are given by these gentlemen, in confirmation of their opinions, which certainly are most strong in favour of the draughts. One says, "it is a specific remedy for the cure of the Asiatic cholera;" another, that it "is a chemical agent which positively neutralizes the morbific poison of cholera;" a third, that it cures "as by enchantment," that it "acts miraculously."

The most striking results seem to have been obtained at Mataro, where the remedy was employed by all the practitioners of the place. Mr. Parkin, who unfortunately has no positive data respecting the epidemic in this town, says, however, that he "understood" that the number affected was upwards of a thousand, and the deaths about sixty.

It is obvious that Mr. Parkin is far from being justified by the result of his own cases, in pronouncing effervescing saline draughts to be the antidote of cholera; and, in reference to the Spanish evidence, in the absence of specific documents, and with the ample experience we had in this country of the *extreme difference* of the epidemic in different places, is it not infinitely more probable that the particular epidemic treated with such happy results by the Spanish physicians, was one naturally mild and tractable, one that would have yielded to the remedial powers of nature, than that the terrible cholera, which in many parts of this country, and in some parts of Spain, produced such havoc, could have been subdued by a few saline draughts? It is impossible to come to any other conclusion. At the same time we are disposed to admit that, in the present state of our knowledge, or rather ignorance, of the pathology of this terrible disease, and in our utter unacquaintance with any means proved to be capable of arresting its progress when manifesting itself in its severe forms, we would much rather trust to the effervescing draughts of Mr. Parkin,* or the cold water of Dr. Shute, than to agents of more potent influence on the animal economy.

In his second Work, Mr. Parkin extends his theory and his antidotal treatment to the yellow fever and dysentery of tropical climates, with the same unction of confidence in their verity and power, and on equally valid grounds. The only facts, however, adduced in support of his doctrines and practice are about a dozen cases of *ague*, and a smaller number of cases of slight dysentery, or diarrhœa, in Spain and England! The cases of ague certainly got well under the use of the saline draughts, and some of them so speedily, and after the failure of other apparently more effective treatment, that we must either admit the power of the remedy in such cases, or find an explanation of the cure in some concomitant circumstances not stated in the documents. We therefore recommend a trial of the practice in our marshy districts. We fear, however, it will be found that the saline draughts are but a feeble substitute for quinine. At any rate, even admitting that they can cure an ague, what warrant have we that they shall cure yellow fever or tropical dysentery? Alas, none; while all reason, and, what is more, all experience, is against the belief that they should do so. Indeed, we feel assured that, when Mr. Parkin has had opportunities—as we have had—of treating the real malignant yellow fever of the West Indies, and the real Asiatic cholera, he will painfully prove the utter powerlessness of his antidote, and look upon his former confidence in it as one of those happy dreams of generous youth which time and the actual world so painfully dissipate.

* Mr. Parkin is, of course, not the individual who first employed this remedy in cholera, it being the ordinary prescription in most cases of gastric irritation whencesoever arising. In a paper in the Med. Gazette, August 25, 1832, Dr. Stevens says "when the stomach is irritable the saline draughts are of great value: . . . this given frequently lessens the irritation, and does more good than any other remedy that I have seen tried in those cases where the stomach is so irritable that it cannot retain the stronger salts."

